# Identification of Glutathione Synthetase as a Therapeutic Target for Cervical Cancer via Combining Bioinformatics and Experimental Validation

**DOI:** 10.1111/jcmm.70968

**Published:** 2025-12-22

**Authors:** Meini Pan, Tingzhuang Yi, Peng Lei, Jiangmi Mo, Jinyan Lan, Yulu Ye, Hongqian Wang, Cheng Yuan, Zhaohe Huang

**Affiliations:** ^1^ Guangxi Medical University Nanning China; ^2^ Department of Oncology Affiliated Hospital of Youjiang Medical University for Nationalities Baise China; ^3^ Guangxi Clinical Medical Research Center for Hepatobiliary Diseases Baise China; ^4^ Department of Radiology Affiliated Hospital of Youjiang Medical University for Nationalities Baise China; ^5^ Youjiang Medical University for Nationalities Baise China; ^6^ Department of Nasopharyngeal Oncology Red Cross Hospital of Yulin City Yulin China; ^7^ Department of Oncology, Yichang Central People's Hospital and The First College of Clinical Medical Science China Three Gorges University Yichang China; ^8^ Tumor Prevention and Treatment Center of Three Gorges University, Cancer Research Institute of Three Gorges University Yichang China; ^9^ Clinical Medical Research Center for Precision Diagnosis and Treatment of Lung Cancer and Management of Advanced Cancer Pain of Hubei Province Yichang China; ^10^ Affiliated Hospital of Youjiang Medical University for Nationalities Baise China

**Keywords:** bioinformatics, cervical cancer, cuproptosis, glutathione synthetase, vorinostat

## Abstract

Cervical cancer remains a leading cause of cancer‐related mortality among women worldwide, posing a severe threat to female health. Previous research indicates that cuproptosis is a copper‐dependent form of regulated cell death, holding potential as a therapeutic avenue. This study aimed to identify and validate Cuproptosis‐Related Genes (CRGs) as biomarkers and therapeutic targets in cervical cancer. Transcriptomic data from TCGA and GTEx databases were analysed alongside curated literature data, leading to the identification of 67 pivotal CRGs. Diagnostic and prognostic models were constructed using machine learning algorithms and LASSO‐Cox regression, respectively. Glutathione synthetase (GSS) was selected for subsequent functional validation in cellular assays. Drug sensitivity analysis, mechanistic investigations and in vivo experiments were conducted to evaluate therapeutic potential. Statistical analyses were performed using R and GraphPad Prism. Our analysis identified GSS as a core gene. Functional experiments showed that GSS promotes cervical cancer cell proliferation and invasion under cuproptosis‐inducing conditions. Drug sensitivity analysis linked GSS to vorinostat, which inhibits tumour growth by suppressing the PI3K/Akt pathway and downregulating GSS. These findings were confirmed in both in vitro and in vivo studies. This study identifies GSS as a key cuproptosis regulator and a promising therapeutic target in cervical cancer, suggesting a novel precision medicine strategy.

## 1 Introduction

Cervical cancer remains one of the most common gynaecological malignancies worldwide and the fourth leading cause of cancer‐related deaths among women, posing a substantial threat to women's health globally [1]. According to 2022 global cancer statistics, approximately 660,000 new cases of cervical cancer were diagnosed, with about 350,000 deaths attributed to the disease, nearly 60% of which occurred in Asia [2, 3, 4]. The current first‐line treatment for advanced cervical cancer includes surgery, radiotherapy or platinum‐based chemotherapy combined with bevacizumab [5, 6]. However, the efficacy of these regimens remains suboptimal for a significant proportion of patients. Notably, the 5‐year survival rate for patients with advanced disease falls below 20%, largely due to frequent distant metastasis and the development of therapy resistance [7].

Routine screening for cervical cancer primarily relies on the ThinPrep Cytology Test (TCT) and high‐risk Human Papillomavirus (HPV) DNA testing. Nevertheless, TCT carries a non‐negligible false‐positive rate, while HPV testing cannot differentiate between persistent and transient infections, often leading to potential over‐treatment [8]. Furthermore, conventional diagnostic approaches exhibit limited capacity to accurately assess tumour heterogeneity [9]. The complexity of metastatic mechanisms and the frequent emergence of drug resistance further complicate clinical treatment. Therefore, the identification of reliable diagnostic biomarkers and effective therapeutic targets is of critical importance for improving detection and treatment outcomes in cervical cancer.

In recent years, breakthroughs in multi‐omics technologies have provided novel perspectives for systematically deciphering the molecular mechanisms of cancer. Genomic studies have revealed associations between somatic mutations in key genes such as PIK3CA, KRAS and PTEN and malignant transformation [10]. Single‐cell transcriptomics has corroborated tumour heterogeneity at the cellular level [11]. Metabolomic analyses further indicate that glutamine‐dependent metabolic reprogramming plays a central role in the progression of cervical cancer [12]. Notably, these technological advances have also accelerated the exploration of novel regulated cell death mechanisms. Among these, cuproptosis is a copper‐dependent form of regulated cell death that holds potential as a therapeutic measure. This mechanism primarily relies on the direct binding of copper ions to mitochondrial TCA cycle proteins, such as FDX1 and DLAT, leading to inactivation of iron–sulfur cluster proteins, induction of proteotoxic stress and ultimately, cell death [13, 14]. In cervical cancer research, multiple lines of evidence support the association between dysregulated copper metabolism and disease pathogenesis. Clinical data indicate significantly elevated serum copper levels in patients [15], while various copper‐related agents, such as LQM402, disulfiram/copper complexes and copper nanoparticles, have demonstrated antitumor potential in experimental models [16, 17, 18, 19]. These findings suggest that cuproptosis may have important implications for the development and treatment of cervical cancer.

This study sought to develop cuproptosis‐based diagnostic and prognostic models for cervical cancer using integrated multi‐omics and machine learning, and to functionally characterise the core gene glutathione synthetase (GSS) to identify potential biomarkers and therapeutic targets.

## 2 Methods

### 2.1 Publicly Available Datasets

The publicly available datasets utilised in this study are summarised in Table S1.

### 2.2 Identification and Functional Annotation of CRGs

Transcriptome data for 306 cervical cancer tissues, along with 3 matched adjacent normal tissues, were retrieved from The Cancer Genome Atlas (TCGA) database. To augment the normal tissue cohort, transcriptomic profiles from 88 additional normal cervical samples were incorporated from the Genotype‐Tissue Expression (GTEx) project. Differential expression analysis was performed using the DESeq2, edgeR and limma packages in R (version 4.3.2), applying a threshold of |log_2_ (fold change)| > 1 and an adjusted *p*‐value < 0.05 for identifying significantly dysregulated genes. Principal Component Analysis (PCA) was conducted using the tinyarray package to validate the separation between cancerous and normal groups. Furthermore, the GSE67522 dataset from the Gene Expression Omnibus (GEO) served as an independent external validation cohort. A consensus set of 390 Cuproptosis‐Related Genes (CRGs) was curated based on combined evidence from CRISPR‐Cas9 knockout screens and prior literature [14, 20], with a False Discovery Rate (FDR) < 0.05. Subsequent functional enrichment analysis included the evaluation of Gene Ontology (GO) terms for biological processes, molecular functions and cellular components using the clusterProfiler package, with significance thresholds set at *p* < 0.05. Additionally, Kyoto Encyclopedia of Genes and Genomes (KEGG) pathway enrichment analysis was performed using the Database for Annotation, Visualisation and Integrated Discovery (DAVID) bioinformatics resource [21, 22], with a statistical significance level of *p* < 0.05.

### 2.3 Machine Learning‐Based Feature Selection and Diagnostic Model Validation

To identify diagnostic biomarkers for cervical cancer, we employed a multi‐step machine learning approach for feature selection. First, Least Absolute Shrinkage and Selection Operator (LASSO) regression with 10‐fold cross‐validation was applied to identify 11 key genes from the pool of 67 cuproptosis‐related Differentially Expressed Genes (DEGs). Second, the Support Vector Machine‐Recursive Feature Elimination (SVM‐RFE) algorithm, utilising a radial basis function kernel, was implemented to iteratively refine the feature set, yielding three genes: BRCA1, CDK1 and GSS. Third, a Random Forest model comprising 1000 trees was constructed to assess gene importance based on the mean decrease in Gini impurity; this analysis selected 22 candidate genes with an importance score > 2. The intersection of the feature sets derived from these three methods was identified using a Venn diagram, which confirmed BRCA1, CDK1 and GSS as the consensus diagnostic gene signature. The diagnostic efficacy of this signature was evaluated by Receiver Operating Characteristic (ROC) curve analysis in both the combined TCGA‐GTEx cohort and an independent validation cohort (GSE67522). Subsequently, a logistic regression‐based nomogram was constructed using the GSE67522 dataset to facilitate clinical prediction.

### 2.4 Construction and Validation of a Cuproptosis‐Related Prognostic Model

A prognostic model based on CRGs was constructed using the glmnet package. Overall survival data and expression profiles of cuproptosis‐related DEGs from the TCGA dataset were integrated to perform LASSO‐Cox regression analysis. The optimal penalty parameter (*λ* = 0.03) was selected via 10‐fold cross‐validation, resulting in the identification of 16 prognostic gene biomarkers. Subsequently, multivariate Cox regression analysis was carried out with the survival package to further refine the model and ascertain genes possessing independent prognostic value. The prognostic performance of the model was assessed using Kaplan–Meier survival analysis, time‐dependent ROC analysis and Decision Curve Analysis (DCA).

### 2.5 Expression Pattern and Functional Enrichment Analysis of GSS

The expression pattern of GSS was evaluated across multiple cohorts, including gynaecological cancers from TCGA and normal tissue controls from the GTEx project, as well as the independent validation cohort GSE67522. To corroborate transcriptional findings at the protein level, Immunohistochemical (IHC) evidence was consulted from the Human Protein Atlas (HPA) database. For functional interpretation, Gene Set Enrichment Analysis (GSEA) was conducted using software (version 3.0), obtained from the GSEA portal.

### 2.6 Drug Sensitivity

To evaluate the potential therapeutic relevance of GSS, we assessed its association with drug sensitivity by performing Spearman correlation analysis using data from the CellMiner database.

### 2.7 Molecular Docking Analysis

The three‐dimensional structure of Vorinostat (also known as SAHA) was retrieved from the PubChem database, and the PDB file of the GSS protein was obtained from the UniProt database. Molecular docking was performed using the AutoDock Vina (version 1.2.5) software, followed by visualisation with PyMOL (version 4.6.0). Two‐dimensional interaction diagrams were generated using LigPlot+ software.

### 2.8 Cell Culture

The human cell lines HeLa (catalogue no. LBH‐210) and SiHa (catalogue no. LBH‐127) were acquired from Lai Bai Ha (Shanghai) Biotechnology Co. Ltd. (Shanghai, China). All cell lines were maintained in their respective recommended media, supplemented with 10% Fetal Bovine Serum (FBS) (Cell‐Box, AUS‐01S‐02) and 1% penicillin–streptomycin (100 U/mL penicillin and 100 μg/mL streptomycin), at 37°C in a humidified atmosphere containing 5% CO_2_. All cell lines were routinely tested to confirm the absence of mycoplasma contamination. Furthermore, their identities were authenticated, and they were confirmed not to be among the list of commonly misidentified cell lines published by the International Cell Line Authentication Committee (ICLAC).

### 2.9 Cell Transfection and Functional Assays

To investigate the functional role of GSS, we established stable GSS‐overexpressing HeLa and SiHa cell lines via transfection with a human GSS plasmid (pCMV3‐SV40‐Pu‐CMV‐cMyc‐GSS; Nanjing Corues Biotechnology). Transfection efficiency was validated by qRT‐PCR at 24 h and Western blot at 48 h post‐transfection. Subsequently, to assess the interaction between GSS and vorinostat under cuproptosis stress, the transfected cells were subjected to a 24 h pretreatment with 1 μM vorinostat. Prior to functional assays, these cells were then exposed to 40 nM Elesclomol and anhydrous Copper chloride (ES‐Cu) complex for 2 h to induce cuproptosis. The ES‐Cu complex was formed by combining ES (MedChemExpress, HY‐12040) and Cu (Macklin, C804817) at a 1:1 M ratio.

Cell proliferation was assessed using CCK‐8 assay (Servicebio, G4103), with absorbance measured at 450 nm after 2 h incubation. DNA synthesis was evaluated by EdU incorporation assay (Beyotime, C0078S), where EdU‐positive cells were counted from five random fields after staining. Cell migration was examined through wound healing assays, with wound closure quantified using ImageJ at 0 and 24 h. Transwell chambers (8 μm pores; Corning, 353097) were used to evaluate migration and invasion capabilities, with Matrigel (MedChemExpress, HY‐K6001) coating applied specifically for invasion assays. Cells that migrated or invaded through the membrane were stained with crystal violet and counted from five random fields. All experiments were conducted with three independent biological replicates. Data are expressed as mean ± SD, and statistical significance was determined using Student's t‐test or one‐way ANOVA (*p* < 0.05).

### 2.10 Patients and Samples

This study utilised five paired Formalin‐Fixed, Paraffin‐Embedded (FFPE) tissue specimens consisting of primary cervical cancer tissues and matched adjacent normal tissues collected > 3 cm from the tumour margin. All specimens were obtained from Yichang Central People's Hospital between January and July 2025 and processed for IHC staining to evaluate GSS protein expression. Patient inclusion required pathological confirmation of primary cervical cancer and no history of radiotherapy, chemotherapy or other anticancer treatments prior to surgical resection.

### 2.11 Quantitative Real‐Time PCR (qRT‐PCR)

Total RNA was extracted from cultured cells using TRIzol reagent (Vazyme, R401‐01). cDNA was synthesised from 1 μg of total RNA using the HiScript II First Strand cDNA Synthesis Kit with gDNA wiper (Vazyme, R223‐01). Quantitative PCR was performed on a Bio‐Rad CFX96 Touch system with ChamQ Universal SYBR qPCR Master Mix (Vazyme, Q311‐02) in 20 μL reaction volumes. The thermal cycling conditions were: initial denaturation at 95°C for 30 s, followed by 40 cycles of 95°C for 10 s and 60°C for 30 s, with a final dissociation curve analysis from 65°C to 95°C. GAPDH was used as the endogenous control, and all samples were analysed in technical triplicates. Relative expression of GSS was calculated using the 2^−ΔΔCT^ method.

All primers were synthesised by Tsingke Biotechnology (Wuhan, China). The primer sequences used were:

GSS: forward 5′‐CAATGCTCTGGTGCTACTGATTG‐3′, reverse 5′‐CAGTAGACGTGCTTCCCAATTCT‐3′

GAPDH: forward 5′‐AGAAGGCTGGGGCTCATTTG‐3′, reverse 5′‐AGGGGCCATCCACAGTCTTC‐3′

### 2.12 Western Blot Analysis

HeLa and SiHa cells were treated with vorinostat (1.0 or 2.5 μM) for 24 h, followed by a 2‐h exposure to 40 nM ES‐Cu. Cells were lysed in RIPA buffer containing PMSF, and protein concentrations were determined by BCA assay. Proteins were separated on 7.5%–12.5% SDS‐polyacrylamide gels and transferred to PVDF membranes. After blocking with 5% skim milk, membranes were incubated overnight at 4°C with primary antibodies against GSS (ABclonal A11557), AKT (ABclonal A22412), p‐AKT Ser473 (ABclonal AP1208), PI3K (ABclonal A23303PM), p‐PI3K p85α (ABclonal AP0427), GAPDH (ABclonal AC054), HSP70 (Abmart T55496), FDX1 (Abmart T510671). Following incubation with HRP‐conjugated secondary antibody (ABclonal, AS061), protein signals were detected using ECL substrate and visualised by chemiluminescence imaging.

### 2.13 Construction of Nude Mouse Xenograft Models

All animal experiments were conducted in accordance with protocols approved by the Animal Ethics Committee of Youjiang Medical University for Nationalities. Four‐week‐old male BALB/c nude mice, supplied by Shanghai SLAC Laboratory Animal Co. Ltd., were housed under specific pathogen‐free conditions. Mice were subcutaneously inoculated in the left dorsal flank with a suspension containing 5 × 10^6^ HeLa cells. When the xenograft tumours reached a palpable volume (approximately 10 days post‐inoculation), the mice were randomly assigned to two experimental groups (*n* = 5 per group). The control group was administered intraperitoneal injections of ES‐Cu (5 mg/kg daily for 5 consecutive days per week, over 2 weeks) co‐treated with a vehicle control (DMSO injected twice weekly for 2 weeks, at a volume equivalent to the Vorinostat injection). The Vorinostat + ES‐Cu group received identical ES‐Cu dosing in combination with Vorinostat (50 mg/kg, administered twice weekly for 2 weeks). Animal health status and body weight were recorded daily. Tumour dimensions were measured regularly with a digital calliper, and tumour volume was calculated according to the formula: (short diameter)^2^ × long diameter/2. At the experimental endpoint, all mice were humanely euthanized, and tumour tissues were excised and weighed. For subsequent histological examination, tumour samples were fixed, paraffin‐embedded, sectioned and subjected to IHC staining. For Western blot analysis, parallel tumour samples were snap‐frozen in liquid nitrogen and maintained at −80°C until processing.

### 2.14 Ethical Approval

The human tissue samples used in this study were approved by the Ethics Committee of Yichang Central People's Hospital. The approval was issued under the committee’s protocol code AF/SC‑07/1.1, with the assigned ethics approval number 2025‐160‐02. All animal experiments were approved by the Animal Ethics Committee of Youjiang Medical University for Nationalities (Ethics approval number: 2024052805).

### 2.15 Statistical Analysis

Statistical analyses were performed using R and GraphPad Prism 10.1.2. Correlations between gene expressions were assessed by Pearson analysis. Group comparisons were conducted using Student's *t*‐test (for two‐group comparisons) or one‐way Analysis of Variance (ANOVA). Statistical significance was defined as *p* < 0.05.

## 3 Results

### 3.1 Identification and Functional Enrichment of CRGs

**FIGURE 1:**
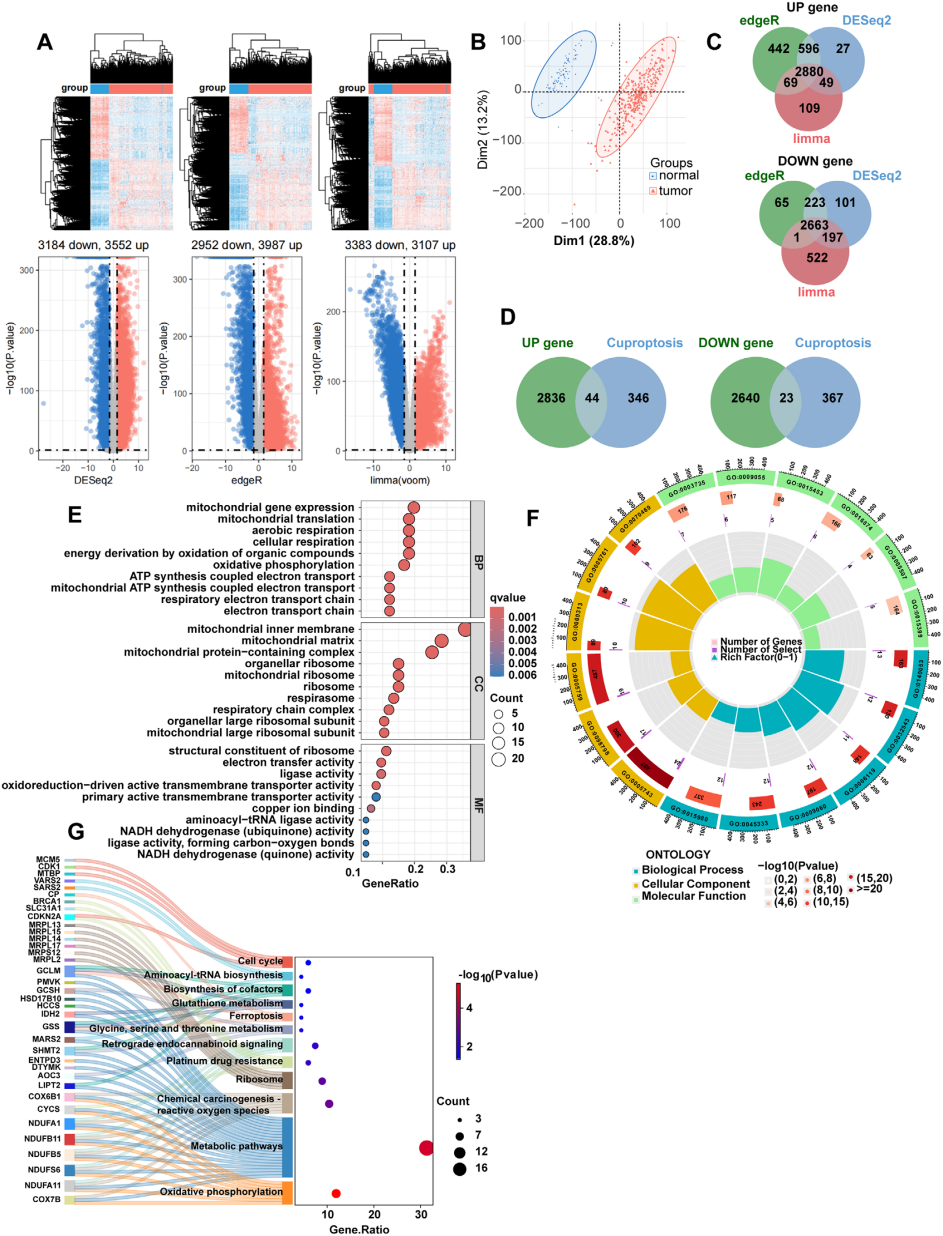
Identification and functional enrichment of CRGs. (A) DEGs in cervical cancer versus normal tissues from the integrated TCGA‐GTEx dataset, as analysed by DESeq2, edgeR and limma. (B) PCA plot distinguishing cervical cancer samples from normal controls. (C) Venn diagram showing the intersection of up‐regulated and down‐regulated DEGs identified by the three algorithms. (D) Venn diagram illustrating the overlap between the consensus DEGs and known CRGs, yielding 67 cuproptosis‐related DEGs. (E,F) GO enrichment analysis of the 67 cuproptosis‐related DEGs. (G) KEGG pathway enrichment analysis of the 67 cuproptosis‐related DEGs. CRGs, cuproptosis‐related genes; DEGs, differentially expressed genes; GO, gene ontology; GTEx, genotype—tissue expression; KEGG, Kyoto Encyclopedia of Genes and Genomes; PCA, principal component analysis; TCGA, The Cancer Genome Atlas.

To systematically investigate the role of cuproptosis in cervical cancer, we first performed transcriptomic differential expression analysis on the TCGA‐ Cervical Squamous Cell Carcinoma and Endocervical Adenocarcinoma (CESC) cohort and the GTEx database. By integrating the results from three algorithms (DESeq2, edgeR and limma), we identified a total of 5543 consistently dysregulated genes, comprising 2880 up‐regulated and 2663 down‐regulated DEGs (Figure 1A–C, Tables S2 and S3). After intersecting with 390 CRGs [14, 20] (Table S4), we obtained 67 cuproptosis‐associated DEGs relevant to cervical cancer, including 44 up‐regulated and 23 down‐regulated genes (Figure 1D, Table S5).

To gain deeper insights into the biological functions of these 67 genes, GO and KEGG enrichment analyses were conducted. GO analysis indicated significant enrichment in biological processes such as ‘mitochondrial translation’ and ‘respiratory chain complex’, as well as in cellular components including the ‘mitochondrial inner membrane’ and ‘mitochondrial matrix’ (Figure 1E,F, Table S6). KEGG pathway analysis revealed that these genes are prominently involved in key pathways related to cellular metabolism, including glutathione metabolism, oxidative phosphorylation, ROS metabolism and platinum drug resistance (Figure 1G, Table S7).

### 3.2 Machine Learning‐Based Screening for Diagnostic Biomarkers in Cervical Cancer

**FIGURE 2:**
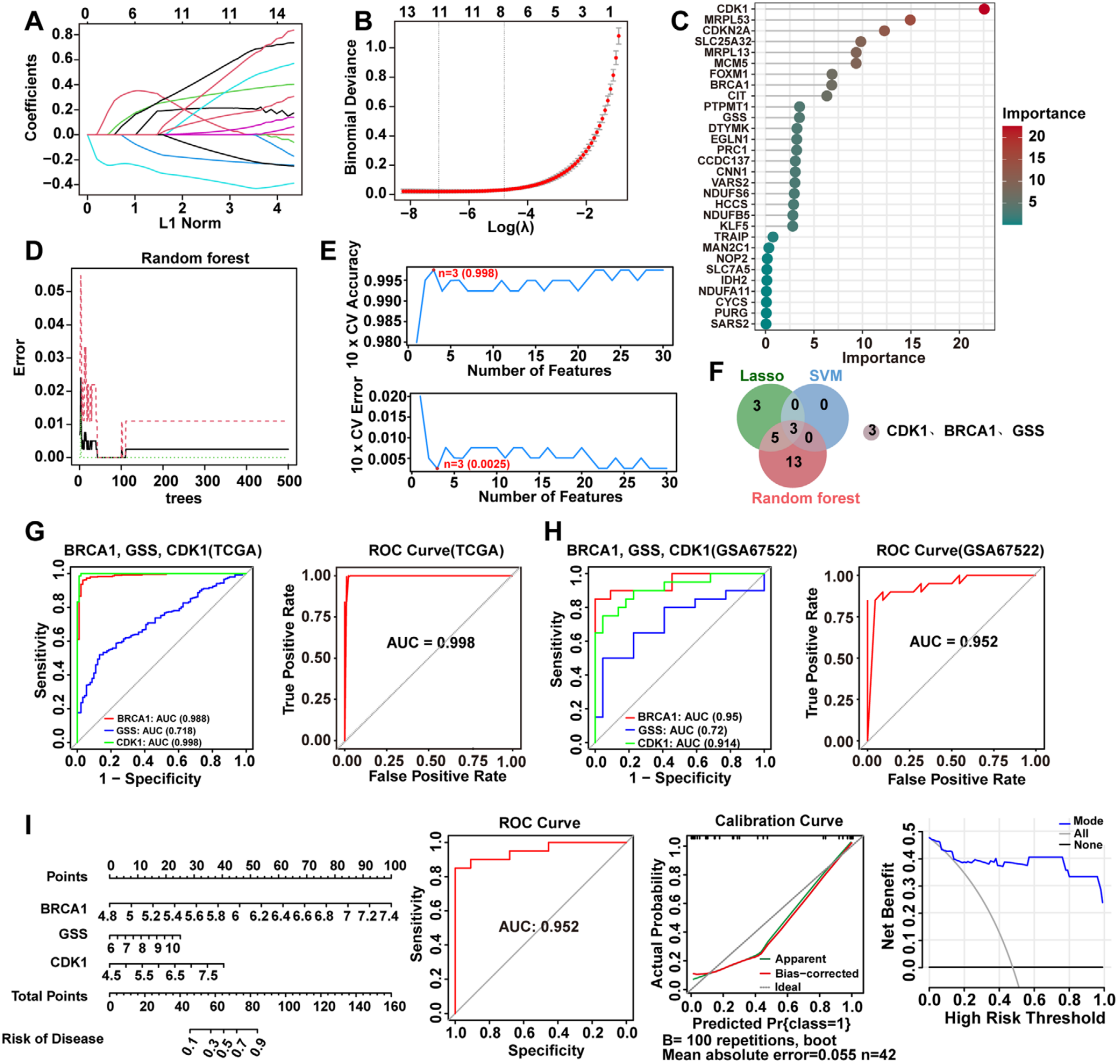
Machine learning‐based screening for diagnostic biomarkers in cervical cancer. (A) LASSO regression coefficient profile. A 10‐fold cross‐validation was used to select the penalty parameter lambda. (B) Binomial deviance versus log(lambda) plot for LASSO regression. The vertical lines indicate the optimal lambda values. (C) Variable importance plot from the Random Forest model. (D) Error rate of the Random Forest model as a function of the number of trees. (E) Support Vector Machine Recursive Feature Elimination (SVM‐RFE) cross‐validation accuracy versus the number of features. The optimal feature set (*n* = 3) is highlighted. (F) Venn diagram identifying the intersection of key genes (CDK1, BRCA1, GSS) selected by LASSO, SVM‐RFE and Random Forest algorithms. (G) Receiver operating characteristic (ROC) curves for the individual genes (BRCA1, GSS, CDK1) and the combined diagnostic model in the TCGA‐GTEx cohort. (H) ROC curves validating the diagnostic performance in the independent GSE67522 cohort. (I) Nomogram for clinical prediction, along with its ROC curve, calibration curve (assessing the agreement between predicted and observed probabilities) and decision curve analysis (evaluating net benefit across different risk thresholds) in the GSE67522 cohort. GTEx, genotype tissue expression; ROC, receiver operating characteristic; SVM‐RFE, support vector machine recursive feature elimination; TCGA, The Cancer Genome Atlas.

To refine the selection of core genes with diagnostic potential from the 67 cuproptosis‐related DEGs, we employed three machine learning algorithms for feature selection. LASSO regression identified 11 genes (Figure 2A,B), the Random Forest algorithm determined 22 genes based on Gini index importance (Figure 2C,D), and the SVM‐RFE algorithm optimised the set to the three most discriminatory genes: BRCA1, CDK1 and GSS (Figure 2E). A Venn diagram confirmed that these three genes represented the common intersection across all three methods (Figure 2F), thus establishing them as the core diagnostic gene signature.

A logistic regression diagnostic model based on this signature demonstrated excellent performance in the internal training set (combined TCGA‐GTEx cohort) (Figure 2G). This performance was robustly validated in an independent external validation set (GSE67522) (Figure 2H). Furthermore, a nomogram incorporating this gene signature was constructed to provide an intuitive clinical prediction tool (Figure 2I). Calibration and decision curve analyses both indicated good accuracy and clinical applicability of the model. These results identify the molecular signature comprising BRCA1, CDK1 and GSS holds potential as a potential diagnostic biomarker for cervical cancer.

### 3.3 Construction and Validation of a Cuproptosis‐Related Prognostic Model

**FIGURE 3:**
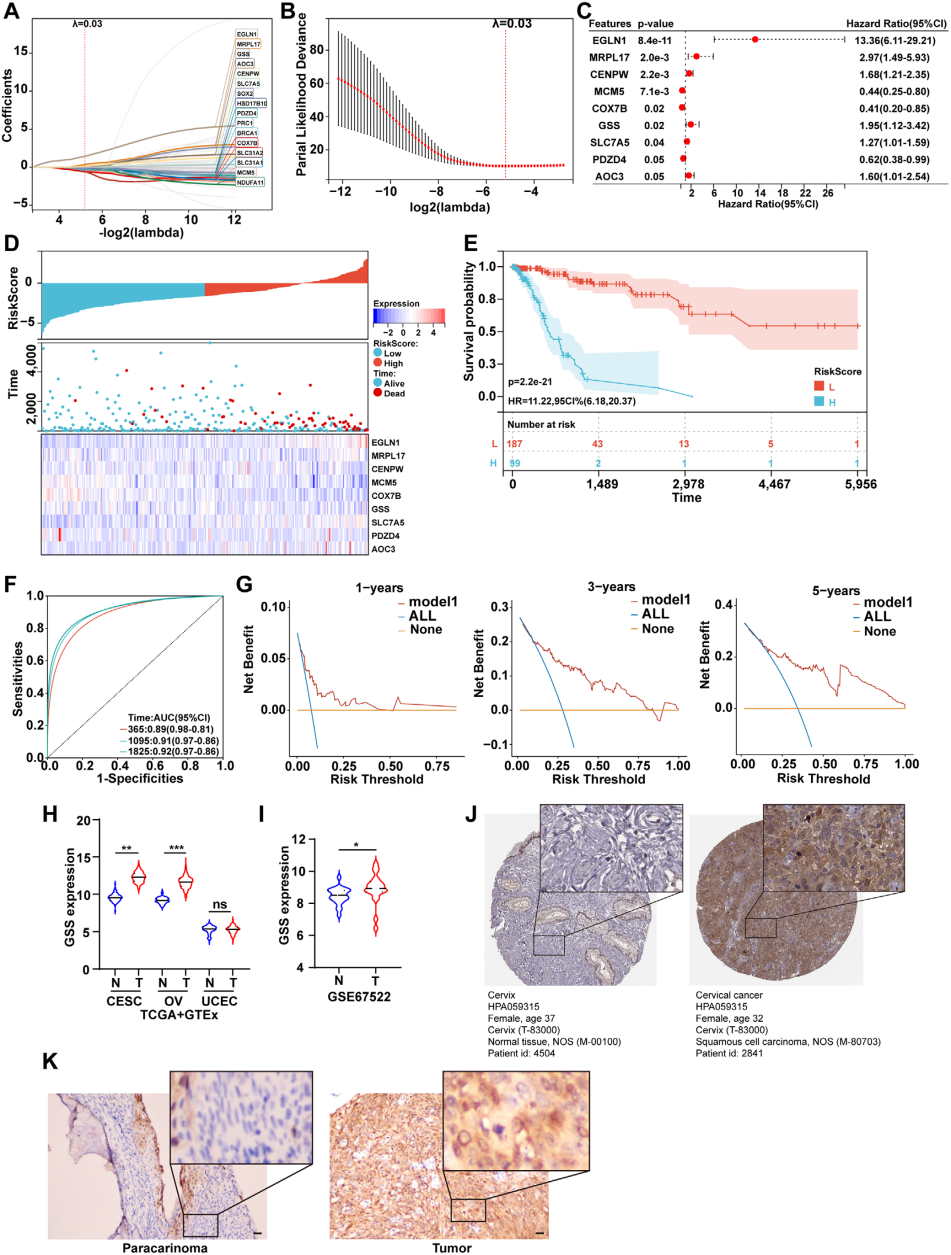
Construction and validation of a cuproptosis‐related prognostic model and upregulation of GSS in cervical cancer. (A) LASSO‐Cox regression identified 16 prognostic genes. (B) Partial likelihood deviance for the LASSO‐Cox model from 10‐fold cross‐validation. (C) Forest plot from the multivariate Cox regression analysis confirming nine genes with independent prognostic value. (D) Correlation analysis between the prognostic risk score, patient survival status, and the expression patterns of the nine model genes. (E) Kaplan–Meier survival curves comparing overall survival between the high‐risk and low‐risk groups. (F) Time‐dependent ROC curves assessing the predictive accuracy of the prognostic model. (G) DCA evaluating the clinical net benefit of the model at 1, 3 and 5 years. (H) GSS mRNA expression levels in cervical squamous cell CESC, OV and UCEC from the TCGA and GTEx databases. (I) Validation of GSS mRNA upregulation in cervical cancer using the independent GSE67522 dataset. (J) IHC images from the HPA database showing GSS protein expression in normal and cervical cancer tissues. (K) Representative IHC images confirming higher GSS protein expression in clinical cervical cancer tissues compared to matched adjacent normal tissues. Scale bar = 50 μm. CESC, cervical squamous cell carcinoma and endocervical adenocarcinoma; DCA, decision curve analysis; GTEx, genotype‐tissue expression; HPA, human protein atlas; IHC, immunohistochemistry; OV, ovarian cancer; ROC, receiver operating characteristic; TCGA, The Cancer Genome Atlas; UCEC, uterine corpus endometrial carcinoma. *p < 0.05; **p < 0.01; ***p < 0.001; ns, not significant.

We next explored the prognostic value of these genes. Using survival data from the TCGA‐CESC cohort and expression levels of the 67 cuproptosis‐related DEGs, a LASSO‐Cox regression analysis was performed, resulting in a 16‐gene prognostic signature (Figure 3A,B). Subsequent multivariate Cox regression analysis refined this to a robust model containing nine genes with independent prognostic value: MCM5, COX7B, PDZD4, EGLN1, MRPL17, CENPW, GSS, SLC7A5 and AOC3 (Figure 3C). Based on their risk coefficients, the first three were defined as protective genes, while the latter six were defined as risk genes. Expression analysis revealed that risk genes were generally up‐regulated in tumour tissues, whereas protective genes tended to be down‐regulated (Figure 3D), consistent with their assigned roles in the model.

Survival analysis confirmed that patients in the high‐risk group, stratified by the model, had a significantly shorter overall survival compared to those in the low‐risk group (Figure 3E). Time‐dependent ROC curves demonstrated reliable accuracy of the model for predicting 3‐year and 5‐year survival rates (Figure 3F,G). These findings support the potential of this cuproptosis‐related signature as an independent prognostic indicator for cervical cancer.

### 3.4 GSS Is Upregulated in Cervical Cancer

Having established diagnostic and prognostic models, we sought to identify a central player. Notably, GSS was the only gene overlapping between the diagnostic three‐gene signature and the prognostic nine‐gene model, suggesting its potentially pivotal role in cervical carcinogenesis. Therefore, GSS was selected for further functional investigation. In the combined TCGA and GTEx data, GSS mRNA levels were significantly higher in cervical cancer and ovarian cancer tissues compared to normal tissues (Figure 3H). This finding was consistently replicated in the independentGSE67522 cohort (Figure 3I). IHC data from the HPA database indicated stronger GSS staining intensity in cervical carcinoma tissues compared to normal cervical tissues (Figure 3J). More importantly, IHC experiments performed on five pairs of clinical cervical cancer and matched adjacent normal tissues confirmed the specific overexpression of GSS protein in tumour tissues (Figure 3K). These consistent findings establish GSS as a potential biomarker for cervical cancer.

### 3.5 GSS Promotes Malignant Phenotypes Under Cuproptosis Induction

**FIGURE 4:**
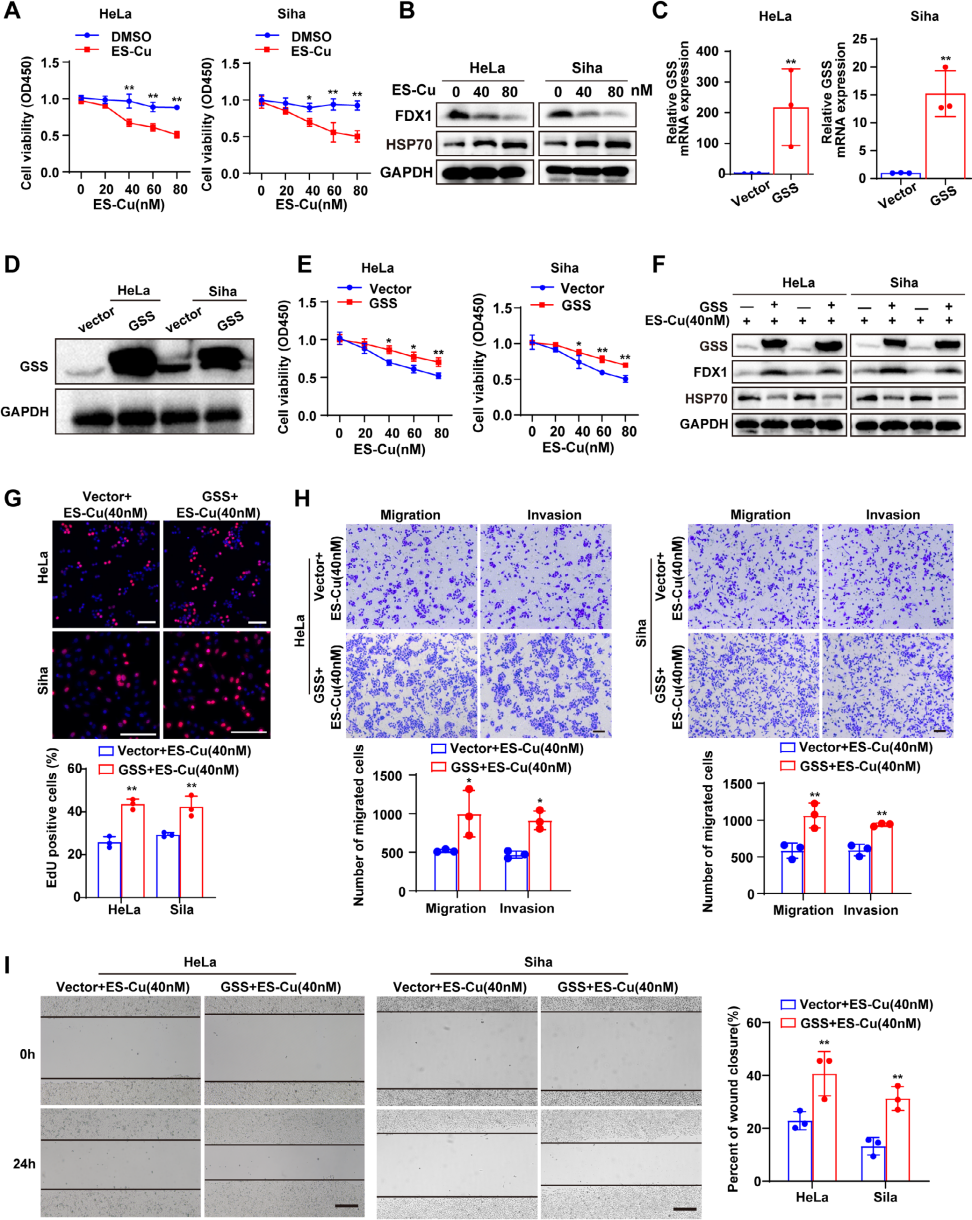
GSS promotes malignant phenotypes under cuproptosis induction. (A) Cell viability of HeLa and SiHa cells treated with increasing concentrations of the copper ionophore ES‐Cu. (B) Western blot analysis showing ES‐Cu‐induced downregulation of FDX1 and upregulation of HSP70. (C) qRT‐PCR analysis confirming the efficiency of GSS overexpression at the mRNA level. (D) Western blot analysis verifying GSS overexpression at the protein level. (E) Cell viability assay showing that GSS overexpression attenuates ES‐Cu‐induced cytotoxicity. (F) Western blot analysis demonstrating that GSS overexpression mitigates the ES‐Cu‐induced alterations in FDX1 and HSP70 protein levels. (G) EdU proliferation assay revealing that GSS overexpression partially rescues cell proliferation capacity under ES‐Cu treatment. Scale bar = 100 μm. (H) Transwell migration and invasion assays indicating that GSS overexpression partially restores migratory and invasive capabilities suppressed by ES‐Cu. Scale bar = 100 μm. (I) Wound healing assay demonstrating that GSS overexpression reverses the ES‐Cu‐induced inhibition of cell migration. Scale bar = 100 μm. ES‐Cu, elesclomol and anhydrous copper chloride; qRT‐PCR, quantitative real‐time polymerase chain reaction. *p < 0.05; **p < 0.01.

To explore the functional role of GSS, we focused our investigation on its role under cuproptosis stress. We first confirmed that the copper ionophore ES‐Cu induced cell death in a concentration‐dependent manner, accompanied by downregulation of the key cuproptosis executor FDX1 and upregulation of HSP70 (Figure 4A,B), indicating successful induction of cuproptosis.

We then successfully established GSS‐overexpressing models in HeLa and SiHa cells (Figure 4C,D). GSS overexpression significantly attenuated ES‐Cu‐induced cytotoxicity (Figure 4E). At the molecular level, GSS overexpression also mitigated the ES‐Cu‐triggered downregulation of FDX1 and upregulation of HSP70 (Figure 4F), indicating that GSS interferes with the cuproptosis process. Subsequent functional assays revealed that under cuproptosis stress, GSS‐overexpressing cells exhibited enhanced proliferative, migratory and invasive capacities compared to control cells (Figure 4G–I). Collectively, these results indicate that GSS promotes tumour progression primarily by suppressing cuproptosis.

### 3.6 Vorinostat Enhances the Antitumor Efficacy of ES‐Cu in Cervical Cancer Cells

**FIGURE 5:**
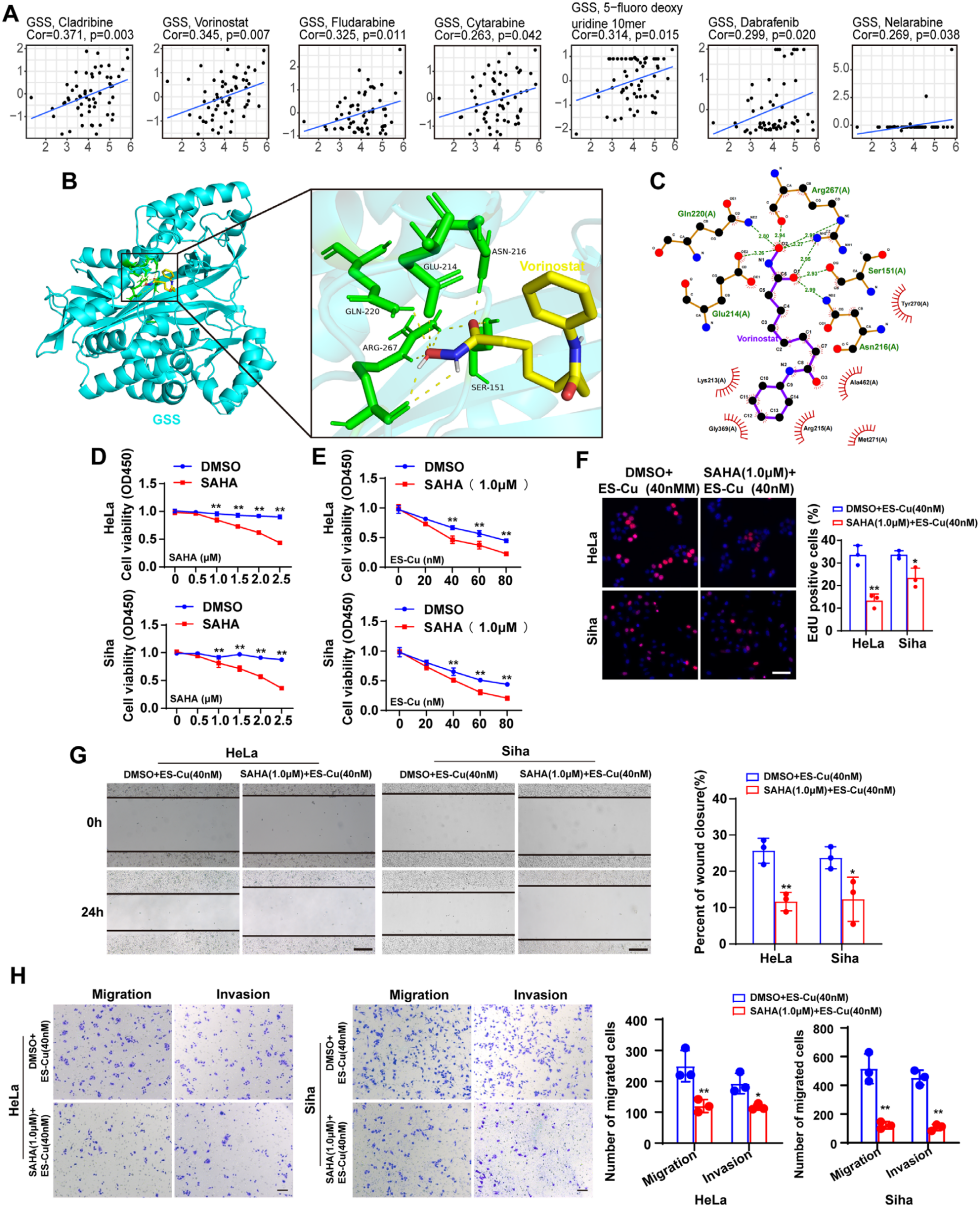
Vorinostat enhances the antitumor efficacy of ES‐Cu in cervical cancer cells. (A) Correlation analysis between GSS expression and drug sensitivity data from the CellMiner database revealed an association with SAHA. (B) Predicted molecular docking pose of SAHA within the substrate‐binding site of the GSS protein. (C) Two‐dimensional interaction diagram of the SAHA‐GSS docking complex. (D) Dose–response curves of HeLa and SiHa cells treated with SAHA alone. (E) Cell viability assay showing enhanced cytotoxicity with the combination of SAHA and ES‐Cu. (F) EdU proliferation assay demonstrating that the SAHA and ES‐Cu combination effectively suppresses proliferation and migration. (G) Wound‐healing assay indicating that the SAHA and ES‐Cu combination markedly inhibits cell proliferation and migration. (H) Transwell migration and invasion assays suggesting that this combination could exert a notably inhibitory effect. ES‐Cu, elesclomol and anhydrous copper chloride; SAHA, vorinostat. *p < 0.05; **p < 0.01; Scale bar = 100 μm.

Based on the above research, we hypothesized that targeting GSS or its regulatory pathway could be an effective therapeutic strategy. Correlation analysis using the CellMiner database revealed a significant association between the histone deacetylase inhibitor Vorinostat and GSS expression (Figure 5A). Considering prior reports that HDAC inhibitors can suppress tumour cell proliferation and invasion [23], we postulated that Vorinostat might be a potential agent targeting GSS function. Moreover, there is a significant correlation between the Vorinostat and GSS expression, and molecular docking also suggests a potential interaction between them (Figure 5B,C).

**FIGURE 6:**
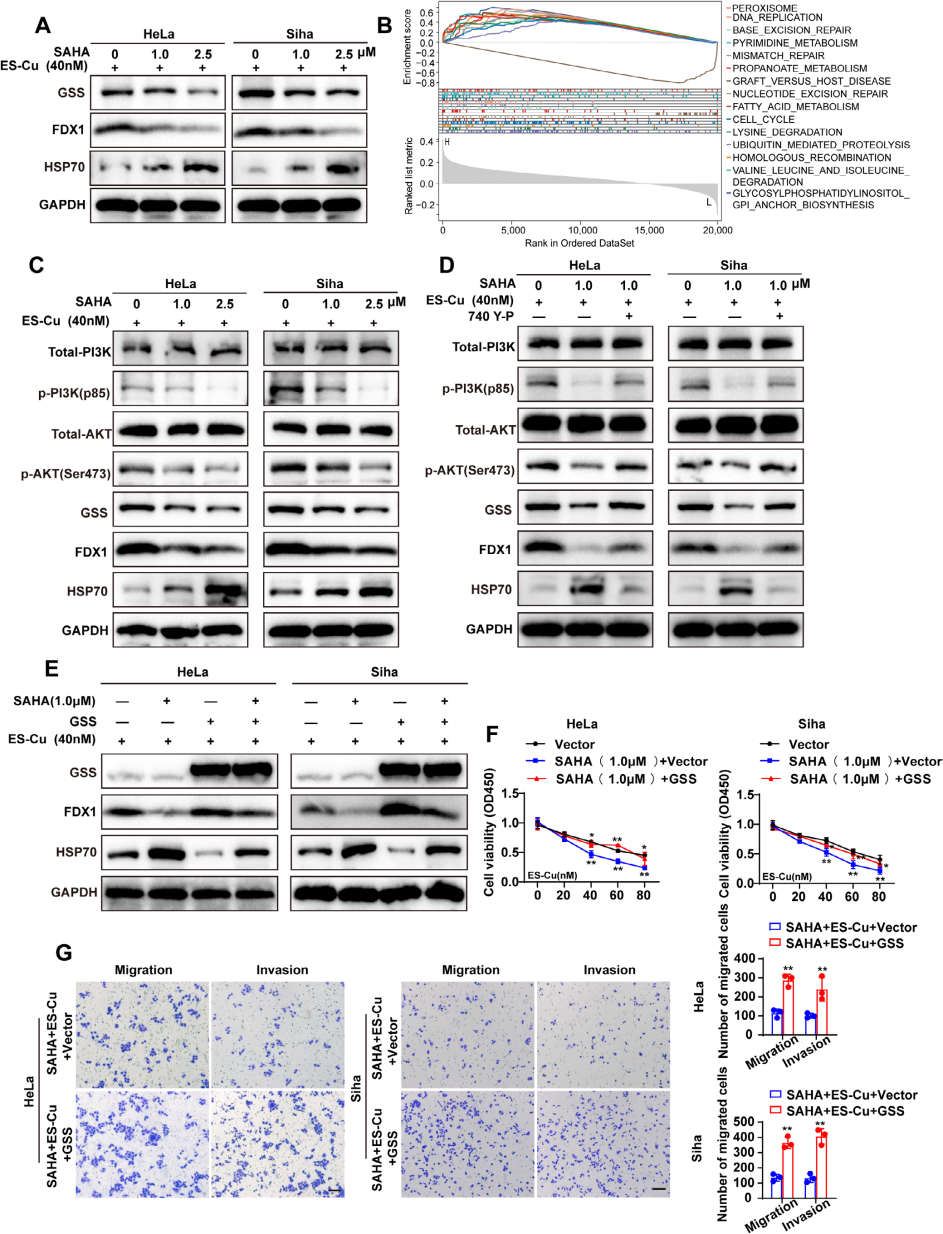
Vorinostat promotes cuproptosis by downregulating GSS via the PI3K/AKT signalling pathway. (A) SAHA and ES‐Cu treatment decreases GSS and FDX1, and increases HSP70. (B) GSEA of pathways associated with GSS expression. (C) SAHA and ES‐Cu reduce PI3K/AKT phosphorylation. (D) 740 Y‐P activates PI3K/AKT phosphorylation, impacting GSS and FDX1. (E) GSS overexpression reverses SAHA's effects on FDX1 and HSP70. (F) GSS overexpression attenuates SAHA and ES‐Cu's impact on cell viability. (G) GSS overexpression attenuates SAHA and ES‐Cu's effects on migration and invasion. ES‐Cu, elesclomol and anhydrous copper chloride; GSEA, gene set enrichment analysis; SAHA, vorinostat. *p < 0.05; **p < 0.01; Scale bar = 100 μm.

We confirmed that Vorinostat alone inhibited cervical cancer cell proliferation in a concentration‐dependent manner (Figure 5D). Subsequent combination treatment experiments showed that Vorinostat pretreatment significantly enhanced the cytotoxic effect of ES‐Cu on cervical cancer cells (Figure 5E). Functionally, this combination regimen most effectively suppressed cell proliferation, migration and invasion (Figure 5F–H). Western blot analysis showed that Vorinostat, in the presence of ES‐Cu, dose‐dependently enhanced the downregulation of FDX1 and upregulation of HSP70, while simultaneously reducing GSS expression (Figure 6A). These findings indicate that Vorinostat sensitises cervical cancer cells to copper‐induced stress and inhibits their proliferative and metastatic capabilities.

### 3.7 Vorinostat Promotes Cuproptosis by Downregulating GSS via the PI3K/Akt Signalling Pathway

To elucidate the molecular mechanism of Vorinostat, we first performed GSEA on signatures associated with high GSS expression. The results showed significant enrichment in various metabolic pathways closely linked to mitochondrial function and cellular redox homeostasis (Figure 6B). As the PI3K/Akt pathway is a key regulator of cell metabolism, survival and death, and prior literature suggests a potential connection with GSS [24], we investigated whether Vorinostat influences GSS through this pathway. Western blot analysis demonstrated that, in the presence of ES‐Cu, Vorinostat treatment dose‐dependently inhibited the phosphorylation of PI3K and AKT, while concurrently downregulating GSS protein expression (Figure 6C). To further detect whether Vorinostat regulates GSS expression through the PI3K/Akt signalling pathway, the PI3K/Akt agonist 740 Y‐P was used. Activation of PI3K/Akt effectively rescued the Vorinostat‐mediated downregulation of GSS (Figure 6D), indicating that Vorinostat reduces GSS expression by inhibiting PI3K/Akt signalling. GSS overexpression partially reversed the Vorinostat‐induced molecular changes characteristic of cuproptosis, namely the downregulation of FDX1 and upregulation of HSP70 (Figure 6E), and partially restored cancer cell proliferation, migration and invasion capacities (Figure 6F,G). The above results indicate that Vorinostat exerts its antitumor effects by inhibiting the PI3K/Akt signalling pathway and downregulating the expression of the GSS protein.

### 3.8 Vorinostat Suppresses Cervical Cancer Growth In Vivo by Targeting the PI3K/Akt/GSS Pathway

**FIGURE 7:**
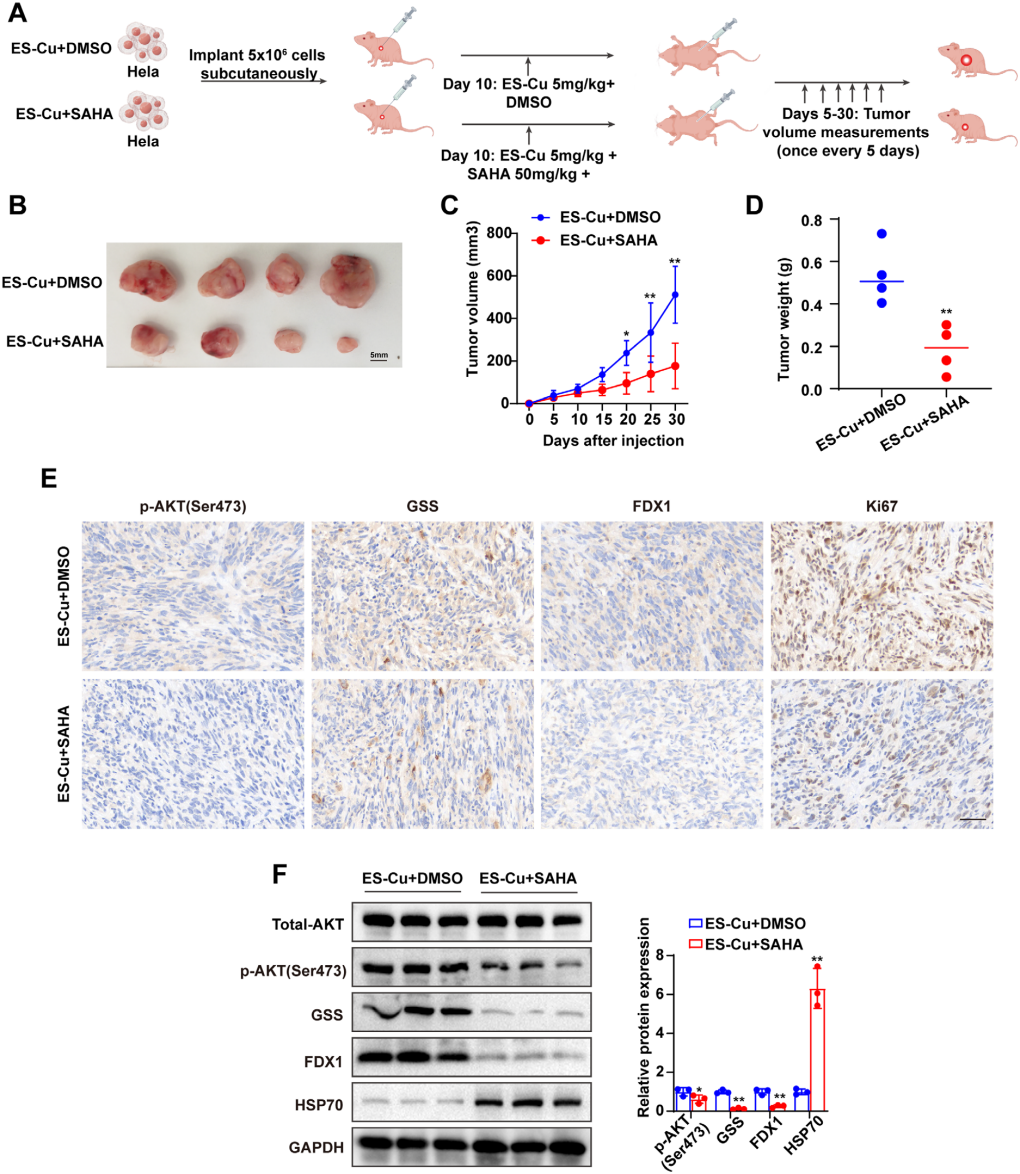
Vorinostat suppresses cervical cancer growth in vivo by targeting the GSS pathway. (A) Establishment of tumour‐bearing mouse model and treatment regimen. (B) Representative images of subcutaneous tumours in mice. Smaller tumour volume in the combination treatment group. Scale bar = 5 mm. (C) Slower tumour growth in the combination treatment group. (D) Significantly reduced tumour weight in the combination treatment group. (E) Representative IHC photomicrographs showing expression of p‐AKT (S473), GSS, FDX1 and Ki67 proteins. Scale bar = 50 μm. (F) Western blot analysis showing reduced AKT phosphorylation, decreased expression of GSS and FDX1, and increased HSP70 in the combination treatment group. IHC, immunohistochemistry. *p < 0.05; **p < 0.01.

To validate the above mechanism in vivo, we established a xenograft model in nude mice using HeLa cells. All tumour‐bearing mice received ES‐Cu to simulate therapeutic stress and were randomly divided into two groups: an ES‐Cu control group and a Vorinostat + ES‐Cu combination therapy group (Figure 7A, the figure was drawn by Figdraw). Consistent with the in vitro findings, combination therapy significantly suppressed tumour growth compared to ES‐Cu alone, as evidenced by smaller tumour volumes (Figure 7B,C) and lower final tumour weights (Figure 7D). Molecular analysis of tumour tissues revealed that the combination group exhibited significantly reduced protein levels of p‐AKT and GSS, accompanied by decreased FDX1 and increased HSP70 (Figure 7F). IHC analysis further corroborated these results, showing a lower positive rate for the proliferation marker Ki‐67, alongside markedly reduced staining intensity for p‐AKT, GSS and FDX1 in the combination treatment group (Figure 7E). These in vivo data demonstrate that Vorinostat inhibits tumour growth by targeting the PI3K/Akt/GSS axis.

## 4 Discussion

This study provides new insights into the role of CRGs in cervical cancer through integrated multi‐omics analyses. We initially identified 67 differentially expressed CRGs in cervical cancer. Functional enrichment analyses revealed that these genes are prominently involved in key pathways related to cellular metabolism, including glutathione metabolism, oxidative phosphorylation, ROS metabolism and platinum drug resistance. Notably, cuproptosis—a newly identified form of cell death—is highly dependent on mitochondrial metabolism [14, 25]. The significant enrichment of oxidative phosphorylation and glutathione metabolism pathways observed in our study aligns closely with the core mechanism of cuproptosis [14]. Furthermore, the Copper ionophore ES exerts antitumor effects by inducing ROS‐mediated cell death [26]. The enrichment of platinum resistance pathways suggests a potential link between resistance to platinum‐based chemotherapeutics and cuproptosis‐related mechanisms. This finding is consistent with previous studies showing that the combination of baicalein and cisplatin promotes apoptosis in cervical cancer cells by inducing cuproptosis [27].

Based on these findings, we further constructed a diagnostic model comprising a three‐gene signature (BRCA1, CDK1 and GSS) using multiple machine learning algorithms. Additionally, via LASSO‐Cox regression, we established a prognostic risk model based on nine genes (MCM5, COX7B, PDZD4, EGLN1, MRPL17, CENPW, GSS, SLC7A5, AOC3), which effectively stratified patients into distinct survival risk groups. It is noteworthy that several of these CRGs have previously been implicated in the malignant progression of cervical cancer. For instance, EGLN1 overexpression has been associated with poorer survival in cervical squamous cell carcinoma [28], and knockdown of SLC7A5 has been shown to significantly suppress the migration and invasion capabilities of cervical cancer cells [29]. Among these genes, GSS stood out as particularly noteworthy due to its simultaneous inclusion in both the diagnostic and prognostic models. Through multiple independent databases and clinical samples, we confirmed that GSS is significantly upregulated in cervical cancer tissues at both the mRNA and protein levels. Survival analysis further indicated that high GSS expression correlates with unfavourable patient outcomes, suggesting its potential as a valuable biomarker in cervical cancer. This observation aligns with findings in other malignancies; for example, Liu et al. [30] reported that GSS is highly expressed in high‐grade glioblastoma and linked to poor prognosis, and similar results have been documented in colorectal cancer studies [31].

Mechanistically, our study revealed a close association between GSS and cuproptosis. We confirmed that the exogenous copper ionophore ES successfully induces cuproptosis in cervical cancer cells, as evidenced by downregulation of the key protein FDX1 and upregulation of HSP70. Previous studies have indicated that ES acts as a copper ionophore, forming a complex with Cu^2+^ that is transported into mitochondria. Excess Cu^2+^ then binds to the lipoylated component of DLAT, leading to aberrant DLAT oligomerization and an increase in insoluble DLAT, ultimately inducing cell death [14, 32, 33]. We further investigated whether GSS influences tumour progression by modulating cuproptosis. Overexpression of GSS significantly attenuated ES–Cu‐induced cell death, reversed the protein‐level changes in FDX1 and HSP70, and enhanced cancer cell proliferation, migration and invasion, indicating that GSS promotes tumour progression primarily by suppressing cuproptosis. As the key rate‐limiting enzyme catalysing glutathione (GSH) synthesis, GSS directly regulates intracellular GSH homeostasis. Aberrant GSH levels have been widely observed in multiple cancer types and are considered a well‐established biomarker [31, 34, 35, 36]. Previous studies have demonstrated that in colorectal cancer, GSS overexpression significantly enhances metabolic flux from cysteine to GSH, thereby promoting tumour growth [31]. Notably, intracellular GSH at high concentrations can directly chelate free copper ions via its thiol group, forming stable GSH–Cu^2+^ complexes. This effectively neutralises the oxidative toxicity of copper ions and prevents their aberrant binding to mitochondrial TCA cycle proteins, thereby averting subsequent iron–sulfur cluster protein degradation and toxic protein aggregation [32, 37]. Multiple studies have also shown that GSH levels are significantly reduced under copper‐overloaded conditions [38, 39], and cells under oxidative stress exhibit increased susceptibility to cuproptosis [40]. Therefore, we speculate that GSS upregulation establishes an efficient GSH–Cu^2+^ buffering system in tumour cells. By maintaining intracellular free copper at sub‐toxic levels, this system delays or inhibits the onset of cuproptosis.

Vorinostat, a Histone Deacetylase (HDAC) inhibitor, has been demonstrated to exert anticancer effects through multiple mechanisms, including inducing cancer cell death, suppressing proliferation and modulating immune responses [41, 42]. Studies have shown that Vorinostat not only activates p21 expression to induce cell cycle arrest and apoptosis [43], but also interferes with tumour cell proliferation, metabolism, and survival by regulating multiple signalling pathways such as NF‐κB, JAK/STAT, PI3K‐AKT and mTOR [44, 45, 46, 47]. In this study, we further elucidate a novel mechanism of Vorinostat in cervical cancer. Our findings demonstrate that Vorinostat significantly suppresses phosphorylation of the PI3K/Akt signalling pathway and downregulates GSS expression, thereby inhibiting tumour progression both in vitro and in vivo. This observation aligns with the report by Xia et al. [46], which indicated that Vorinostat inhibits cervical cancer cell growth via the PI3K/Akt pathway. Furthermore, other studies have reported that Vorinostat reduces the levels of enzymes involved in GSH biosynthesis and increases expression of GSTP1, an enzyme that promotes the conjugation of GSH with cisplatin. Consistent with these findings, intracellular GSH levels decreased in a Vorinostat concentration‐dependent manner [48]. In summary, our findings not only identify a novel target of Vorinostat but also provide a theoretical foundation for its clinical application in cervical cancer treatment.

## 5 Conclusion

In conclusion, our study underscores the significant clinical potential of cuproptosis‐related mechanisms and the key regulator GSS in advancing the diagnosis, prognostic stratification and therapeutic strategies for cervical cancer. The integration of GSS into clinical models may enhance early detection accuracy and provide a robust framework for assessing patient survival risks, thereby facilitating more personalised treatment. Furthermore, the identification of GSS as a central player in modulating cuproptosis sensitivity provides promising avenues for novel therapeutic interventions, as demonstrated by the efficacy of Vorinostat in targeting this pathway.

To translate these findings into clinical practice and improve patient outcomes, further investigations and interdisciplinary collaboration are essential. This necessitates concerted efforts among clinical oncologists, gynaecologists, clinical immunologists, clinical pathologists, cancer immunobiologists, translational immunobiologists, biomedical engineers, regenerative medicine specialists, personalised medicine specialists, cellular and molecular medicine specialists, translational medicine specialists, experimental medicine specialists, medical and translational biotechnologists, cell‐based vaccine researchers, medical laboratory scientists, basic medical scientists, disease‐specific cellular and molecular biomarker specialists and health system coordinators.

## Author Contributions

**Meini Pan:** data curation (equal), formal analysis (equal), funding acquisition (equal), methodology (equal), writing – original draft (equal). **Tingzhuang Yi:** data curation (equal), formal analysis (equal), funding acquisition (equal), methodology (equal), writing – original draft (equal). **Peng Lei:** investigation (equal), software (equal), validation (equal). **Jiangmi Mo:** investigation (equal), software (equal), validation (equal). **Jinyan Lan:** investigation (equal), validation (equal). **Yulu Ye:** investigation (equal), validation (equal). **Hongqian Wang:** formal analysis (equal). **Cheng Yuan:** conceptualization (equal), project administration (equal), supervision (equal), writing – review and editing (supporting). **Zhaohe Huang:** conceptualization (equal), project administration (equal), supervision (equal), writing – review and editing (supporting).

## Funding

This work was supported by the Project for Enhancing Young and Middle‐aged Teacher’s Research Basis Ability in Colleges of Guangxi (Grant No. 2025KY0554), the National Natural Science Foundation of China (Grant No. 82460475), and the Noncommunicable Chronic Diseases‐National Science and Technology Major Project (Grant No. 2024ZD0524600).

## Consent

All co‐authors consented for publication.

## Conflicts of Interest

The authors declare no conflicts of interest.

## Supporting information


**TABLE S1:** Public databases used in this study.
**TABLE S2:** The complete list of 2880 up‐regulated DEGs identified in the TCGA‐CESC cohort versus GTEx.
**TABLE S3:** The complete list of 2663 down‐regulated DEGs identified in the TCGA‐CESC cohort versus GTEx.
**TABLE S4:** The compiled list of 390 CRGs from published sources.
**TABLE S5:** The final set of 67 cuproptosis‐associated DEGs (44 up‐ and 23 down‐regulated) in cervical cancer.
**TABLE S6:** Full results of the GO enrichment analysis for the 67 cuproptosis‐associated DEGs.
**TABLE S7:** Full results of the KEGG pathway enrichment analysis for the 67 cuproptosis‐associated DEGs.

## Data Availability

All data for this study is available from corresponding authors if required.
